# Evaluation of PIMA™® Point of Care Technology for CD4 T Cell Enumeration in Kenya

**DOI:** 10.1371/journal.pone.0067612

**Published:** 2013-06-25

**Authors:** Matilu Mwau, Ferdinard Adungo, Silvia Kadima, Ephantus Njagi, Carolyne Kirwaye, Najma Salim Abubakr, Lucy Atsieno Okubi, Mary Waihenya, Judi Lusike, Jackson Hungu

**Affiliations:** 1 Kenya Medical Research Institute, Nairobi, Kenya; 2 Clinton Health Access Initiative, Nairobi, Kenya; 3 Deloitte Consulting LLC, Nairobi, Kenya; Blood Systems Research Institute, United States of America

## Abstract

CD4+ T cell enumeration is used to determine eligibility for antiretroviral therapy (ART) and to monitor the immune status of HIV-positive patients; however, many patients do not have access to this essential diagnostic test. Introducing point of care (POC) testing may improve access. We have evaluated Alere’s PIMA™, one such POC device, against conventional CD4+ testing platforms to determine its performance and validity for use in Kenya. In our hands, Alere PIMA™ had a coefficient of variability of 10.3% and of repeatability of 175.6 cells/µl. It differed from both the BD FACSCalibur™ (r^2^ = 0.762, mean bias −64.8 cells/µl), and the BD FACSCount™ (r^2^ = 0.874, mean bias 7.8 cells/µl). When compared to the FACSCalibur™ at a cutoff of 350 cells/µl, it had a sensitivity of 89.6% and a specificity of 86.7% in those aged 5 years and over (Kw = 0.7566). With the BD FACSCount™, it had a sensitivity of 79.4% and a specificity of 83.4% in those aged 5 years and over (Kw = 0.7790). The device also differed from PARTEC Cyflow™ (r^2^ = 0.781, mean bias −24.2 cells/µl) and GUAVA™ (r^2^ = 0.658, mean bias −0.3 cells/µl) platforms, which are used in some facilities in Kenya. We conclude that with refinement, Alere PIMA™ technology has potential benefits for HIV-positive patients. This study highlights the difficulty in selecting the most appropriate reference technology for technical evaluations.

## Introduction

CD4+ T cell quantification by flow cytometry is considered accurate, precise and reproducible [1], [2]. A CD4+ T cell count is positively linked to long-term survival rates and indicates the level of immunosuppression[3]–[5]. During testing, patients older than five years of age are given an absolute CD4+ T cell count, which is determined as CD4+ cells/µL of blood. For patients five years old and younger, it is necessary to measure the percentage of CD4+ T cells among all lymphocytes [6].

Despite the fact that CD4+ T cell enumeration is essential in the initiation of antiretroviral therapy and the monitoring of care and treatment in Kenya, many HIV-positive patients still do not have reliable access to these services [7], [8]. CD4 testing is often only available at centralized laboratories with significant infrastructure investments and highly skilled laboratory technicians [7]. Laboratory networking has improved access somewhat, but sample transportation networks are still so poor that many patients are unable to access adequate and necessary CD4 testing.

Currently, CD4 testing using flow cytometry technology is available in many central and regional laboratories in Kenya using BD FACSCount™ or BD FACSCalibur™ (Becton Dickinson, Franklin Lakes, NJ, USA), PARTEC Cyflow™ (Partec GmbH, Munster, Germany), or GUAVA™ (EMD Millipore Corporation, Billerica, MA, USA) platforms. Unfortunately, this combination of technologies is insufficient to provide CD4 testing to all patients who need them. Long turn-around times for tests sent to central laboratories delay clinical decisions and put a considerable burden on patients. Conventional CD4 testing requires samples be transported in complicated and inefficient sample transportation networks and over long, rough roads. These transportation networks are limited and expensive, and are often compounded by the difficulties of short sample stability.

Fortunately, high quality Point-Of-Care (POC) diagnostic technologies are promising solutions to critical CD4 testing needs in areas without existing laboratory capacity or easy access to conventional CD4 testing laboratories[9]–[13]. For example, HIV rapid diagnostic tests have increased patient access to HIV diagnosis in the last decade [14]. POC diagnostic technologies offer several advantages in that they are generally small, robust, relatively low cost, require little infrastructure, and require minimum technical skills. Additionally, replacement devices can be sent to a site immediately to sidestep POC device downtime. CD4 POC technologies can ease human resource capacity shortages and testing backlogs at central laboratories by diverting samples that are customarily referred. Most importantly, CD4 POC testing can allow doctors to make treatment decisions faster and achieve significant improvements in patient health outcomes.

POC diagnostic technologies for HIV testing, viral load determination and CD4 enumeration are in various stages of development. PIMA™ (Alere Inc., Waltham, MA) is one such portable bench top fixed volume cytometer for CD4+ T cell enumeration. It uses cartridges, which, according to the manufacturer, have a long shelf life and are stable in conditions of high heat and humidity. It can be operated using external power or an onboard rechargeable battery, which has an eight-hour life. Although this technology is being used in some countries and has generated results comparable to already existing technologies[15]–[19], we wanted to evaluate the Alere PIMA™ CD4 POC technology in Kenya.

## Methods

### Study Setting

This study was done in nine health facilities offering CD4+ T cell enumeration: Kenya Medical Research Institute-Centre for Biotechnology Research and Development Nairobi (KEMRI-CBRD), Matibabu Foundation’s Ukwala, University of Nairobi-Department of Immunology, St. Luke’s Hospital Kaloleni, Mariakani District Hospital, Rongo District Hospital, Andersen Medical Centre, Kitale District Hospital and Alupe District Hospital. The sites provided from a range of settings: rural versus peri-urban or urban, high volume versus low volume sites, different day temperatures (ranging from 18°C to 38°C) and different humidity levels (ranging 25%–75%). The sites used different testing platforms (BD FACSCount™, PARTEC Cyflow™, GUAVA™ and BD FACSCalibur™) for routine CD4+ T enumeration.

### Study Participants

All patients attending the selected facilities for HIV treatment and care were eligible for this study. Only patients who provided written informed consent were enrolled in the study. In the case of children below 18 years of age, a parent or legal guardian made the decision regarding participation and gave written informed consent. A total of 1,549 patients were recruited from the nine study sites. Data on gender was available for 1,482 patients: 984 (66.3%) were female, while 498 (33.7%) were male. The median age was 36 years (range 1–75 years old) with an approximately normal distribution; 68 patients were aged 18 years or less. At least 49.7% of the patients were on antiretroviral therapy. Qualified and trained laboratory technicians conducted all tests.

### Study Design

In this methods comparison study, venous and capillary blood specimens were collected consecutively from all eligible patients presenting at the health clinics included in the study who agreed to participate in the study through informed consent. Demographic data, CD4+ T cell count, date of clinic visit and antiretroviral (ARV) use were all recorded in a structured questionnaire and entered into an Access database. The data from all testing sites was similarly uploaded remotely to a central server. This study was reviewed and approved by the Kenya Medical Research Institute Ethical Review Committee (Protocol No. SSC1880). For all patients under the age of 18 years, a parent or guardian gave written informed consent. Children between the ages of 13 and 17 years also gave assent. Patients were only provided with CD4+ T cell results obtained from the conventional CD4 testing platforms for further clinical management.

### Laboratory Procedures

CD4 testing using each of the available devices was done according to manufacturers’ instructions. Whole blood was used for the BD FACSCount™, the BD FACSCalibur™, the PARTEC Cyflow™, the Alere PIMA™ device and the GUAVA™ platforms. Capillary blood was also used for the Alere PIMA™ device. Both internal quality assurance (IQA) and external quality assurance (EQA) schemes were implemented for the platforms according to manufacturers’ instructions and existing individual laboratory protocols. Facility flow cytometers in the sites are enrolled in various External Quality Assurance Schemes. Kenya Medical Research Institute, St. Luke’s Hospital Kaloleni, Andersen Medical Centre, University of Nairobi and Mariakani District Hospital are enrolled with the National Reference Laboratory Quality Assurance Scheme. Matibabu Foundation, Kitale District Hospital, Rongo District Hospital and Alupe District Hospital are enrolled with the Western Province External Quality Assurance Scheme (WEPEQAS). Additionally, all technologists are trained annually in good laboratory practice, immunophenotyping for flow cytometry, reverse pipetting, biosafety and good phlebotomy practice. All devices used in this study passed EQA prior to study commencement and all staff conducting testing were trained to perform routine IQA. Control cartridges (both high and low) were run on the PIMA device every morning before tests. PIMA devices reporting errors were not used for this work.

### Data Analysis

The results of the evaluation were analyzed using standard statistical methods. The absolute CD4+ T Cell counts derived from Alere PIMA™ device were compared with those derived from existing technologies by calculating the coefficient of determination (r^2^) and conducting regression analysis using STATA v. 12 for Mac OSX. To determine interchangeability between the device and exisiting platforms, Bland-Altman analysis and Lin's concordance correlation coefficient (rhoC) were used. For the former analysis, the bias was defined as the mean difference between two methods. The limits of agreement (LoA) between the methods compared were calculated as the mean ±1.96 Standard Deviations (SD) of the differences between the results obtained. Confidence intervals for bias and for limits of agreement were calculated using formulae previously described by Bland and Altman [20]. The x axis on each Bland-Altman plot was the average value of the two methods while the y axis was the difference between the two methods. For Lin’s concordance correlation, the coefficient of determination was derived by squaring the coefficient of determination r and was used to quantify the percentage variation of the dependent variable that could be attributed to the variations in the independent variable of the correlation equation. Both the coefficient of variation and the coefficient of repeatability were also calculated for the Alere PIMA™ device. The coefficient of variation was used to define the instrument precision and was calculated as the (standard deviation×100)/mean of a set of repeated measurements on one sample using one instrument. The coefficient of repeatability, a measure of test-retest precision was defined as the variation in duplicate measurements for several samples, taken by a single technician using the same instrument under the same conditions. It was calculated as 1.96 times the standard deviation of the differences between the two measurements. To determine the effect of the platform under evaluation on eligibility of patients for antiretroviral therapy, Cohen’s weighted kappa statistic (Kw) was used.

## Results

Since we evaluated Alere PIMA™ in the context of existing technologies. The coefficients of repeatability and the mean bias between Alere PIMA™ and existing technologies are all summarized in Table 1.

**Table 1:** Summary of coefficients of determination and mean bias for all comparisons between instruments.

Platform	Sample	Comparison	BD FACSCalibur(whole blood)	BD FACSCount(whole blood)	GUAVA(whole blood)	PARTE Cyflow	Alere PIMA Capillary samples
Alere PIMA	Whole Blood samples	Coefficient of determination	0.762	0.874	0.681	0.852	0.821
		Bias (95% Limits of Agreement)	−64.8 (−332.5, 203.0)	7.8 (−168.9, 184.4)	23.9 (−329.6, 281.9)	−10.0 (261.4, 241.4)	−7.7 (−236.2, 220.7)
	Capillary blood samples	Coefficient of determination	–	0.738	0.658	–	–
		Bias (95% Limits of Agreement)	–	8.6 (−235.4, 252.7)	−0.3 (−315.0, 315.6)	–	–
BD FACSCount	Whole Blood samples	Coefficient of determination	0.885	–	–	–	–
		Bias (95% Limits of Agreement)	−76.5 (−316.0, 163.0)	–	–	–	–

**Figure 1:**
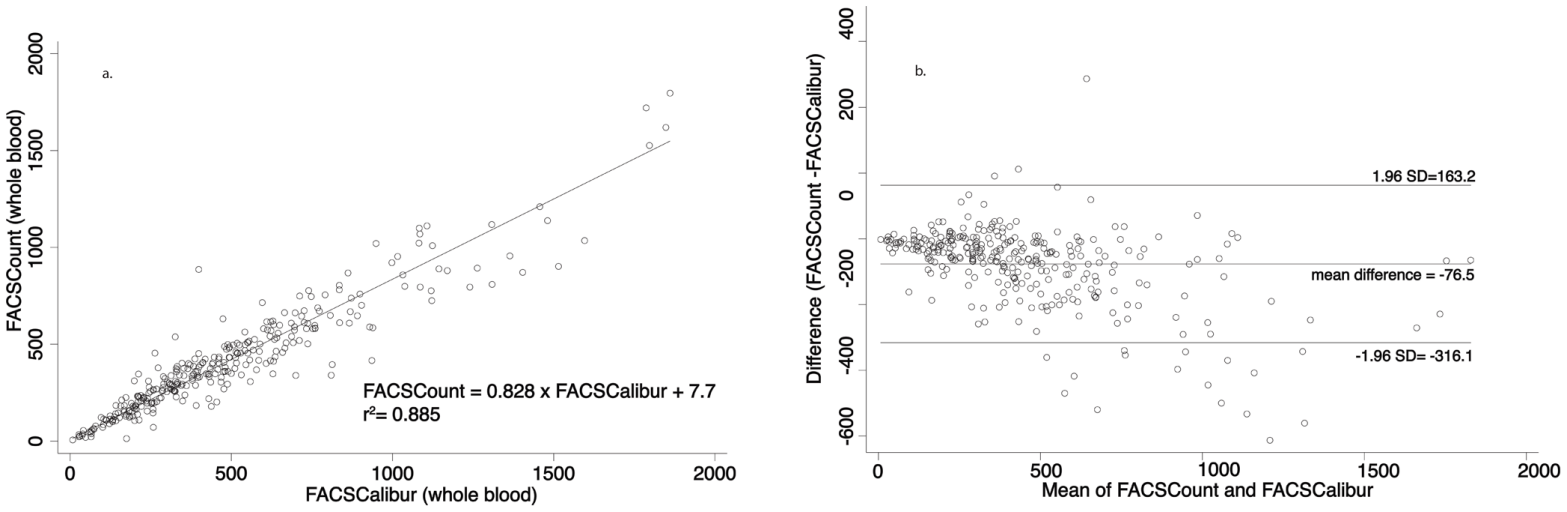
Linear regression analysis (a) and Bland-Altman analysis (b) of absolute CD4+ T lymphocyte counts between FACSCalibur and FACSCount using whole blood.

We began by evaluating the correlation of the platform in most common usage (BD FACSCount™) with the gold standard (BD FACSCalibur™) using blood samples from 312 patients collected at KEMRI CBRD. The coefficient of determination (r^2^) was 0.885. The mean bias between the two platforms was −76.5 cells/µl (95% LoA: −316.0, 163.0). Figs. 1a. & 1b. provide concordance and limits of agreement plots for this relationship.

**Figure 2:**
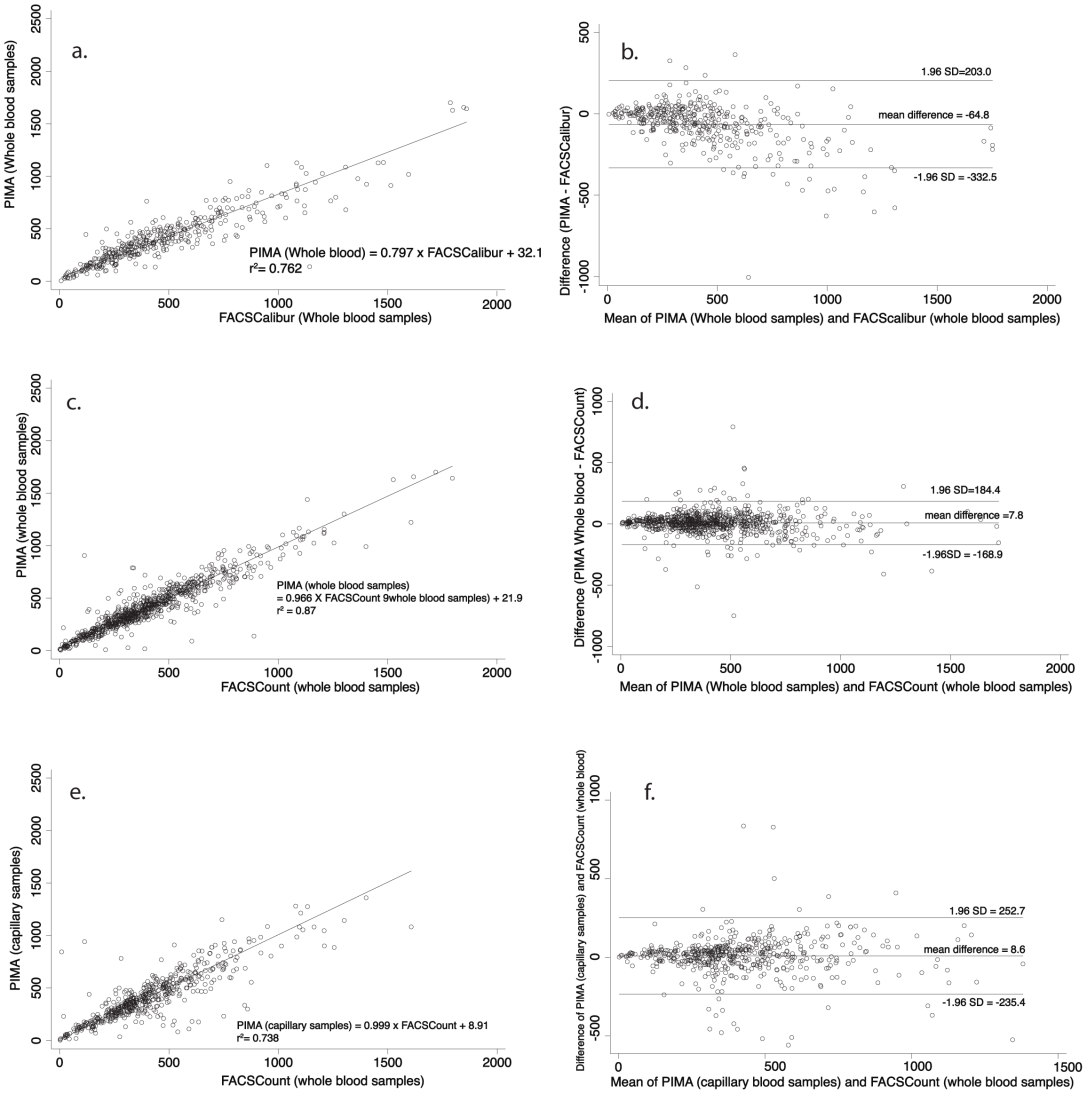
Linear regression analysis and Bland-Altman analysis of absolute CD4+ T lymphocyte counts between PIMA and FACSCalibur using whole blood samples (a & b), PIMA and FACSCount using whole blood samples (c & d), and PIMA (capillary blood samples) and FACSCount (whole blood samples) (e & f) respectively.

We then compared Alere PIMA™ against the gold standard BD FACSCalibur™. A total of 396 patients’ whole blood specimens (339 from KEMRI CBRD and 57 from the University of Nairobi Immunology Laboratories) were tested on the BD FACSCalibur™ and the Alere PIMA™ platforms. The coefficient of determination (r^2^) in this case was 0.762. The mean bias between the two platforms was −64.8 cells/µl (95% LoA −332.5, 203.0). Figs. 2a. & 2b. provide concordance and limits of agreement plots for this comparison.

Thereafter, we compared the Alere PIMA™ with the BD FACSCount™ platform, using whole blood for each. This evaluation used 822 samples of whole EDTA-anti-coagulated blood. 204 samples were drawn from patients visiting Alupe Sub District Hospital, 313 from KEMRI CBRD, 167 from Kitale District Hospital 35 from Mariakani District Hospital and 103 from Rongo District Hospital. As shown in Figs. 2a. & 2d., the coefficient of determination (r^2^) was 0.874, with a mean difference between the two platforms of 7.8 cells/µl (95% LoA −168.9, 184.4).

According to manufacturer instructions, Alere PIMA™ will mainly use capillary blood samples. We were interested in finding out how capillary blood samples on Alere PIMA™ compare with whole blood tests on the same platform and other platforms.

Of the 822 patients enrolled for the comparison between Alere PIMA™ and BD FACSCount™, 521 provided an additional capillary blood specimen for the Alere PIMA™. Of these, Alupe Sub District Hospital provided 206, Kitale District Hospital provided 170, Mariakani District Hospital provided 39 and Rongo District Hospital provided 106 samples. An analysis of this data revealed a concordance coefficient of determination (r^2^) of 0.738. In this approach the mean difference was 8.6 cells/µl (95% LoA −235.4, 252.7). Figs. 2e. & 2f. are a graphical representation of these findings.

**Figure 3:**
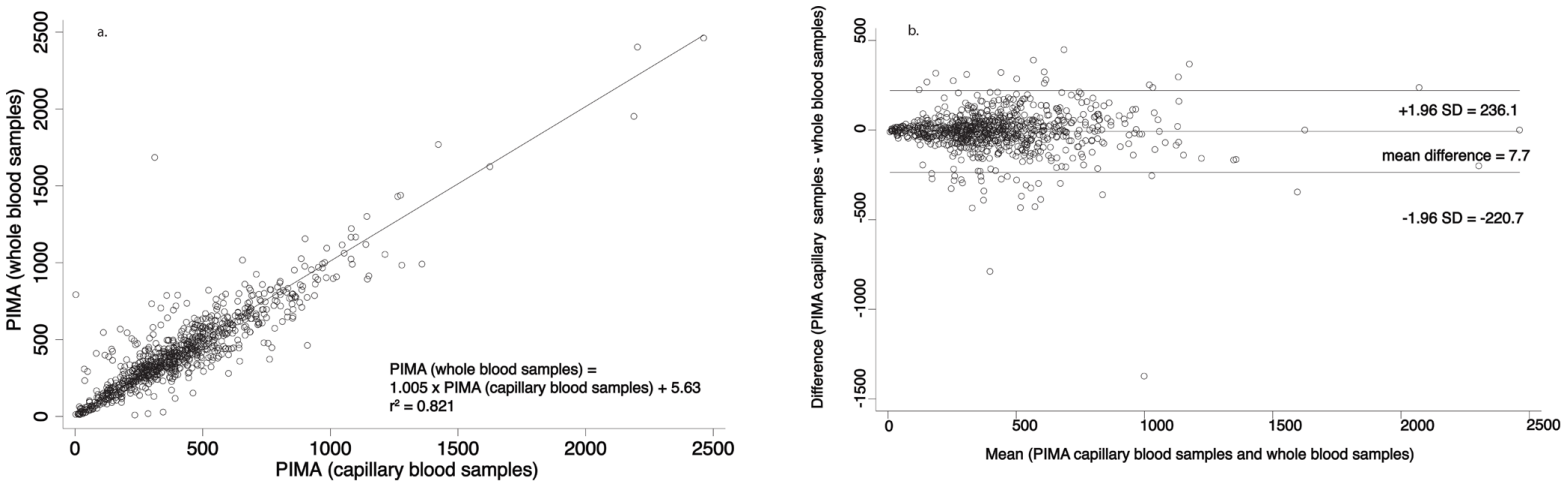
Linear regression analysis and Bland-Altman analysis of absolute CD4+ T lymphocyte counts between PIMA (whole blood samples) and PIMA (capillary blood samples).

When whole blood was compared with capillary blood from the same patient on the same Alere PIMA™ machine, the results were as follows: The mean bias was −7.7 cells/µl (95% LoA −236.2, 220.7). The coefficient of determination (r^2^) was 0.821 (Figs. 3a. & 3b.).

The precision of the Alere PIMA™ platform was determined by running the same sample 16 times using cartridges from the same batch. The mean CD4 count from that sample was 275.9 cells/µl, with a standard deviation of 28.5 cells/µl. The coefficient of variation was therefore 10.3%. To understand the reproducibility of results using the Alere PIMA™ platform, All 211 patients enrolled in the study from Alupe Sub District Hospital provided blood in EDTA tubes each of which was tested twice, on the same Alere PIMA™ device. The mean difference between the tests was 6.9 cells/µl, with a coefficient of repeatability of 175.6 cells/µl.

In comparison, blood collected from 197 patients at Alupe Sub District Hospital was tested using the BD FACSCount™ platform to determine the coefficient of repeatability. The mean difference on the device was 2.2 cells/µl, and the coefficient of repeatability was 66.0 cells/µl.

In Kenya, the Ministry of Health has expressed a desire to identify one point of care platform that can be used interchangeably with all the existing technologies. To that end, we compared the Alere PIMA™ to additional CD4 testing technologies to determine interchangeability with various other platforms.

In a comparison using capillary blood for Alere PIMA™ and whole blood for PARTEC Cyflow™ comparison, the coefficient of determination (r^2^) was 0.852 while the mean bias between these two platforms was −10.0 cells/µl (95% LoA −261.4, 241.4, n = 162). When whole blood specimens (n = 407; 166 patient samples from Andersen Medical Centre and 241 patient samples from University of Nairobi Immunology Department) were used for both platforms the coefficient of determination (r^2^) was 0.781, while the mean bias between these two CD4 enumeration technologies was −24.2 (95% CI −277.6–229.3) cells/µl.

Alere PIMA™ was assessed against the GUAVA™ platform with the former using capillary blood samples and the latter whole blood samples in 176 patients (165 from ACK St Luke’s Hospital Kaloleni and 11 from Andersen Medical Centre). In this exercise, the coefficient of determination was determined to be 0.681. The mean bias between the machines was 23.9 (95% LoA −329.6, 281.9). When whole blood was used in this comparison for both platforms, the coefficient of determination (r^2^) was 0.658. The mean difference was −0.3 (95% LoA −315.0, 315.6, n = 191) cells/µl.

Finally, we assessed whether the sample type might affect the CD4 test result on the Alere PIMA™ platform. 840 samples of each type were compared. r^2^ was 0.821. The mean bias between whole blood and capillary blood samples when tested with the PIMA device was 7.7 cells/µl (95% LoA −220.7, 236.1).

In Kenya, HIV+ patients are eligible for ART initiation when their CD4+ T cell count falls to or below 350 cells/µl. We, therefore, used this threshold to determine the sensitivity and specificity of PIMA™ using either the FACSCalibur™ (which is the gold standard) or the BD FACSCount™ (which is the most commonly available platform).

When compared to the FACSCalibur™, Alere PIMA™ had a sensitivity of 89.6% and a specificity of 86.7% in those aged 5 years and over (n = 389, Kw = 0.7566 ). On the other hand, when compared with the BD FACSCount™, Alere PIMA™ had a sensitivity of 79.4% and a specificity of 83.4% in those aged 5 years and over (n = 813, Kw = 0.7790; Table 2).

**Table 2:** Sensitivity and specificity of Alere PIMA™ using BD FACSCalibur™ as the gold standard test.

			FACSCalibur™ (whole blood)		
			≤350	>350	
PIMA™ (whole blood)		≤350	148	30	Sensitivity 89.7%
		>350	17	201	Specificity 92.2%

When we lowered the threshold to 200 cells/µl, the sensitivity of Alere PIMA™ was 86.7% while the specificity was 94.12% when compared with FACSCalibur™ (n = 389 people aged 5 or older, Kw = 0.7619). Against the FACSCount, the sensitivity and specificity were 83.0% and 98.2% respectively (n = 813, Kw = 0.8422, Table 3).

**Table 3:** Sensitivity and specificity of Alere PIMA™ using BD FACSCount™ as the gold standard test.

		FACSCount™ (whole blood)			
		≤350	>350		
PIMA™ (capillary blood)	≤350	189	37		Sensitivity 79.4%
	>350	49	246		Specificity 83.4%

To contextualize these findings, we compared patient eligibility for ART using BD FACSCount™ with BD FACSCalibur as the gold standard at a threshold of 350 cells/µl. The BD FACSCount™ had a sensitivity of 93.8% and a specificity of 82.4% in those aged 5 years or older (n = 305, Kw = 0.7528). When this threshold was dropped to 200 cells/µl, the sensitivity and specificity became 94.4% and 93.2% respectively (n = 305, Kw = 0.8040).

Finally, we compared the ART eligibility classification rates between the Alere Pima™ and two additional CD4 testing technologies at a threshold of 350 cells/cells/µl (Table 3). With GUAVA™, Kw was 0.7630 (n = 189) while for PARTEC Cyflow™, it was 0.7875 (n = 400).

## Discussion

By the end of 2011, Kenya had approximately 500,000 adults and 40,000 children on ART and, therefore, in need of routine immunological and virological monitoring. HIV testing and ART are available at thousands of health care facilities nationwide, but CD4+ T cell enumeration for ART initiation eligibility and immunological monitoring is, unfortunately, available in only about 200 out of the more than 8000 health care facilities. Laboratory sample referral networking is well established in a few areas, but for a majority of patients, access to CD4 testing is a challenge. In fact, although health care facilities are able to provide ART and monitor clinical outcomes and the side effects of medication, they are often unable to routinely initiate patients on ART due to the inability to access CD4+ T cell enumeration services.

Many of the existing technologies are expensive and not readily accessible. If a new point of care technology can provide reliable and accurate results, and is interchangeable with existing platforms, then it can be used not only to reduce costs and improve access but also to standardize the testing service. The Kenya Ministry of Health believes that it can benefit from economies of scale in a standardized test environment.

It should be pointed out that, by their very nature, point of care tests may not provide the same sensitivity, specificity and accuracy as the “gold standard” reference tests. The trade off is that they can provide access to a CD4 T Cell enumeration service where none exists.

To set the pace for this evaluation, we sought to determine whether the commonest CD4 enumeration platform (BD FACSCount™) in Kenya is interchangeable with the gold standard (BD FACSCalibur™). The coefficient of determination suggested that the agreement between the two platforms was less than perfect. In fact, with a mean bias of 76.5 cells/µl, these platforms are not interchangeable. Differences between the two platforms have been reported in the literature, and may be partially explained by the fact that the BD FACSCalibur™ allows for some subjectivity when laboratory technicians manually gate the CD4+ T cell population.

These differences are clinically important regardless of whether the threshold for ART initiation is set at 350 cells/µl or at 200 cells/µl. If the BD FACSCalibur™ is to be considered the most accurate CD4+ T cell enumeration platform available in Kenya, then our data suggests significant misclassification of patients by the BD FACSCount™. Many patients who are currently on ART due to their CD4 result using the BD FACSCount™ would not have been initiated on ART when tested with the BD FACSCalibur™. We recommend that patients remain faithful to one platform during their care.

When evaluated for precision, Alere PIMA™ gave a coefficient of variability of 10.3%. This level of precision has been reported before [21]. In our opinion, this precision is less than desirable, and much lower than that reported for either the BD FACSCalibur™ or the BD FACSCount™ [22].

There was minimal bias using either capillary blood or whole blood samples on Alere PIMA™. This is a positive finding, suggesting that this platform can be used in a variety of settings. Additional, the reproducibility of results using whole blood on the same Alere PIMA™ platform was very high, another encouraging finding.

Previous studies have reported that the Alere PIMA™ device is comparable and interchangeable with the existing CD4 enumeration platforms[17], [18], [21], [23]–[26]. In our hands, we found no such thing. None of the platforms were interchangeable with the Alere PIMA™ platform. For instance, the sensitivity and specificity of Alere PIMA™ was 83.1% and 92.2% respectively when BD FACSCalibur™ was used as the gold standard and 79.4% and 83.4% respectively when BD FACSCount™ was considered as the gold standard. Even when compared with GUAVA™, Cohen’s kappa was 0.7630 while for PARTEC Cyflow™, it was 0.7875.

In our hands, Alere PIMA™ was not as reliable as previously published literature suggests [18], [21], [26]. For example, the Alere PIMA™ and BD FACSCount™ coefficients of repeatability were very different, at 179.2 cells/µl and 67.4 cells/µl respectively. It appears that tests on the BD FACSCount™ are more repeatable. Alere PIMA™ technology will need to undergo further refinement to improve repeatability.

From our findings, PIMA tended to misclassify patients regardless of the platform used for comparison. This misclassification was generally in favor of undertreatment, and it ranged from 10.4% (17/389) when compared with the BD FACSCalibur™ to 15.3% (59/385) when compared with the BD FACSCount™. This would suggest that if Alere PIMA™ technology is put to widespread use, patients who genuinely need treatment may not qualify for ART, probably only getting ART when already very sick. This is a concern that can be resolved by further improvement of the technology.

For this evaluation laboratory technicians were trained for up to 2 hours on the use of the Alere PIMA™ device. Even with minimal training, the laboratory technicians delivered quality results. It is conceivable that if and when the Alere PIMA™ is deployed in lower levels of health care facilities, the operators would likely be nurses, nurse aides or less technically skilled health care workers. Evaluating this device in the field with such operators would yield additional useful performance information.

The Alere PIMA™ has characteristics of particular interest for use in small health centers located in resource-limited settings. For example, it uses a rechargeable battery rather than require constant electricity. With a turnaround time of 20 minutes per test, the Alere PIMA™ can run up to 20 tests per day on venous or capillary blood. Furthermore, the Alere PIMA™ can store up to 1,000 tests and prints results on a thermal paper roll for recording in patient charts. Using this POC device, personnel can conduct CD4 testing with minimal technical training at remote health facilities. Finally, the Alere PIMA™ reagents have a long shelf life and do not require refrigeration.

### Conclusion

We conclude that with additional refinement, the potential benefits of the Alere PIMA™ technology for HIV-positive patients cannot be exaggerated. It can expand access to CD4 testing, particularly in rural settings whose needs are currently unmet by existing laboratory testing networks. Additionally, this study highlights the difficulty in selecting the most appropriate ‘gold standard’ or reference technology for technical evaluations.
